# Supplementary material for: Novel Amphiphilic Polyfluorene-Graft-(Polymethacrylic Acid) Brushes: Synthesis, Conformation, and Self-Assembly
Source: Polymers (Basel). 2021 Dec 17;13(24):4429. doi: 10.3390/polym13244429 (PMC8708525; doi:10.3390/polym13244429)
Supplement: Supplementary file 1 [file polymers-13-04429-s001.zip › polymers-1494419-supplementary.pdf]

Novel amphiphilic polyfluorene-graft-(polymethacrylic acid) brushes: synthesis, conformation and self-assembly

Maria Simonova*, Dmitry Ilgach, Ksenia Kaskevich, Maria Nepomnyashaya, Larisa Litvinova, Alexander Filippov and Alexander Yakimansky

Institute of Macromolecular Compounds of the Russian Academy of Sciences, Bolshoy pr., 31, Saint Petersburg 199004, Russia; ilgach@yahoo.com (D.I.); mariasimonova1983@mail.ru (M.S.); kaskevich-ksenia@yandex.ru (K.K.); marinepom@mail.ru (M.N.); larissa_litvinova@hotmail.com (L.L.); afil@imc.macro.ru (A.F.); yakimansky@yahoo.com (A.Y.).

* Correspondence: mariasimonova1983@mail.ru; Tel.: +7-812-328-4102 (M.S.)

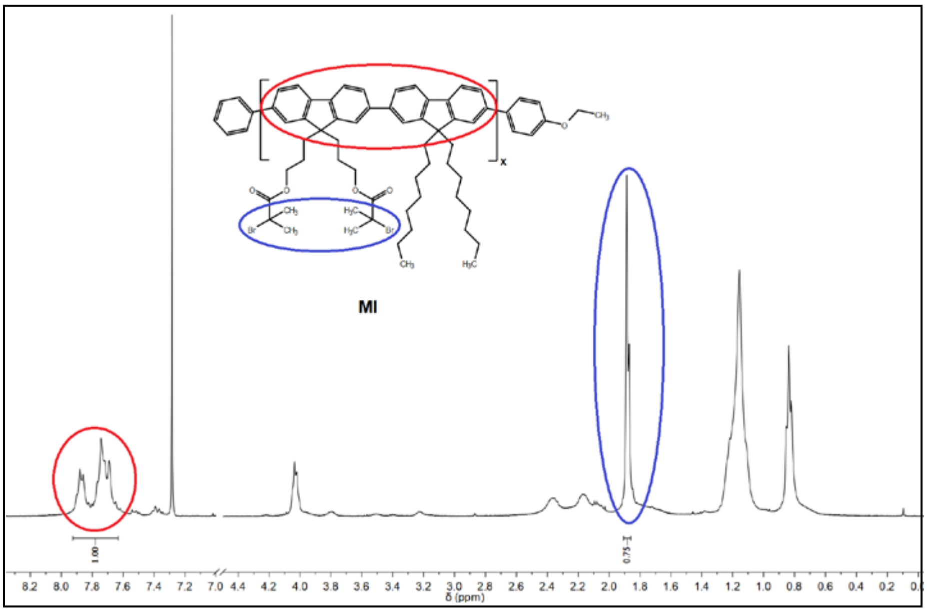

(**a**)

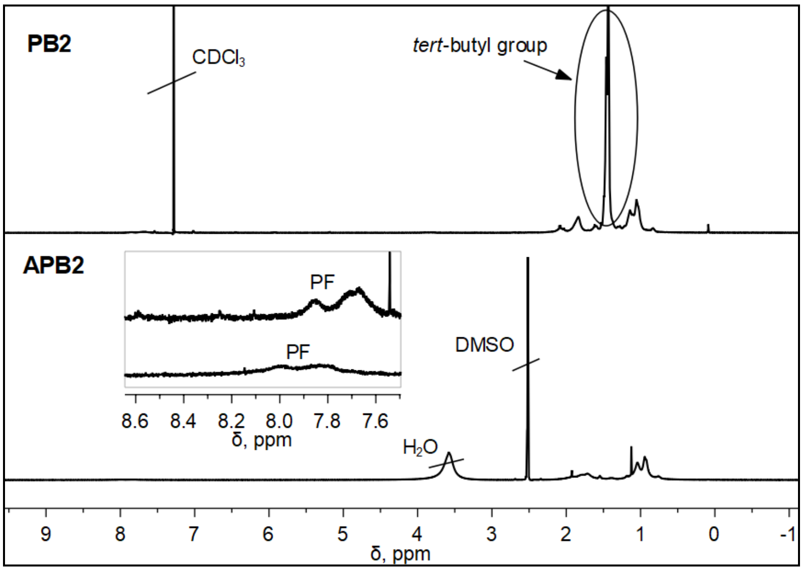

(**b**)

Figure S1. NMR spectra of macroinitiator (**a**), of PB2 and APB2 (**b**).

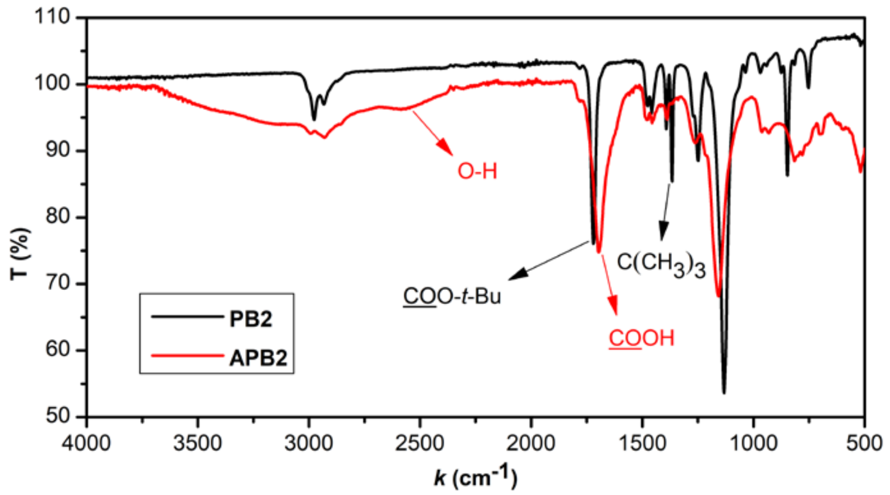

Figure S2. IR spectrum of APB2

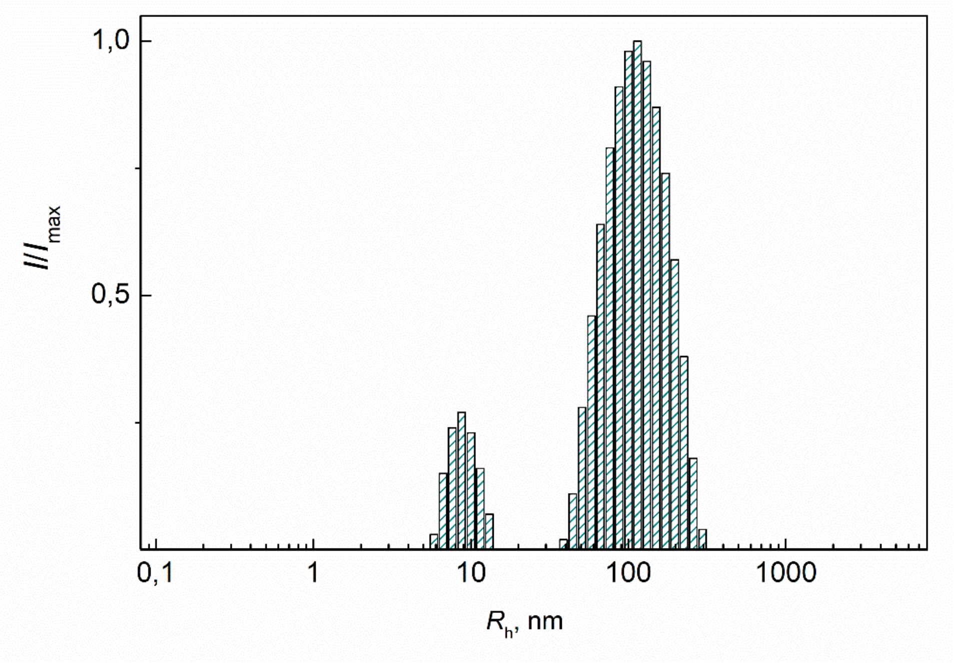

Figure S3. Hydrodynamic radii distribution for MI solution at concentration c = 0.0044 g·cm-3 in chloroform. I_{max} is maximum intensity of scattered light at given concentration.

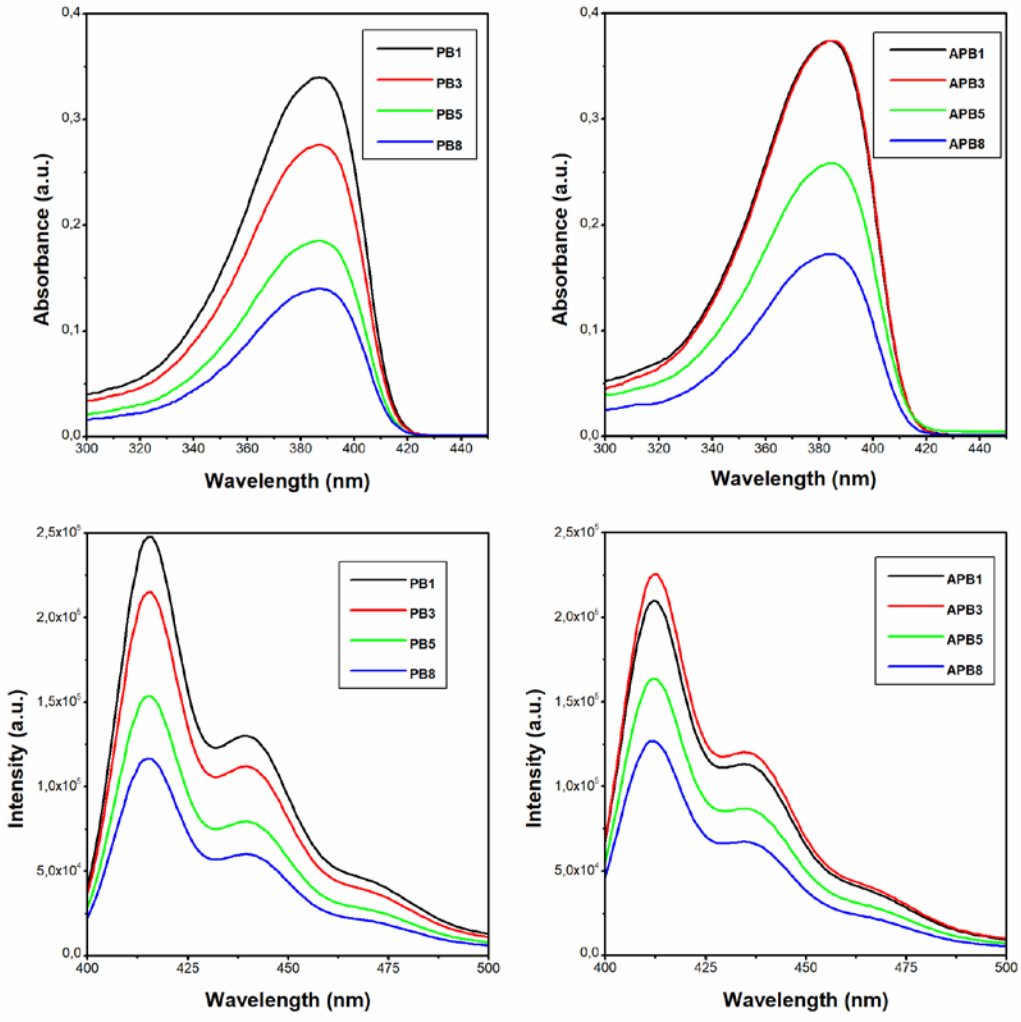

Figure S4. Absorption and luminescence spectra of polymer brushes PB and APB

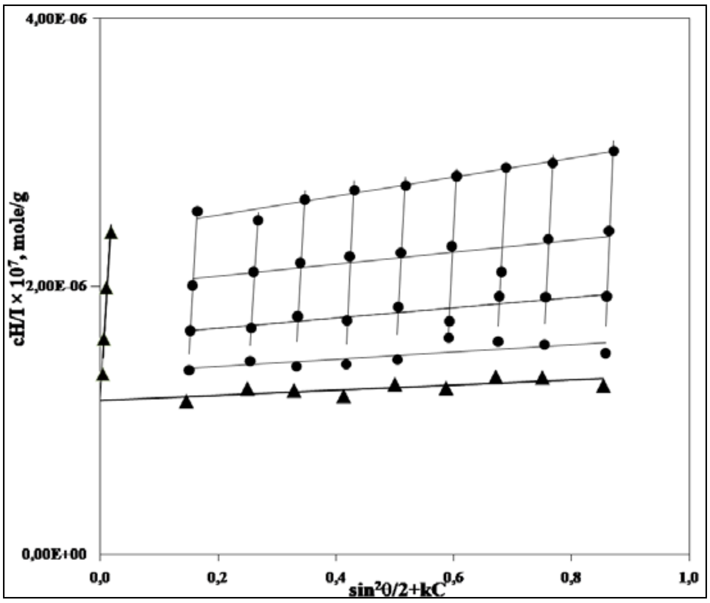

Figure S5. Zimm plot for APB3 in ethanol
